# Pregnancy and infant loss: a survey of families’ experiences in Ontario Canada

**DOI:** 10.1186/s12884-019-2270-2

**Published:** 2019-04-16

**Authors:** Jo Watson, Anne Simmonds, Michelle La Fontaine, Megan E. Fockler

**Affiliations:** 1 Pregnancy and Infant Loss (PAIL) Network, Women and Babies Program, Sunnybrook Health Sciences Centre, 2075 Bayview Avenue, Toronto, Ontario Canada; 2 Lawrence S. Bloomberg Faculty of Nursing, University of Toronto, Toronto, Ontario Canada

**Keywords:** Perinatal loss, Stigma, Miscarriage, Stillbirth, Infant death, Pregnancy loss, Neonatal death, Perinatal care, Ontario, Healthcare professionals

## Abstract

**Background:**

Pregnancy and infant loss has a pervasive impact on families, health systems, and communities. During and after loss, compassionate, individualized, and skilled support from professionals and organizations is important, but often lacking. Historically, little has been known about how families in Ontario access existing care and supports around the time of their loss and their experiences of receiving such care.

**Methods:**

An online cross-sectional survey, including both closed-ended multiple choice questions and one open-ended question, was completed by 596 people in Ontario, Canada relating to their experiences of care and support following pregnancy loss and infant death. Quantitative data were analyzed descriptively using frequency distributions. Responses to the one open-ended question were thematically analyzed using a qualitative inductive approach.

**Results:**

The majority of families told us that around the time of their loss, they felt they were not adequately informed, supported and cared for by healthcare professionals, and that their healthcare provider lacked the skills needed to care for them. Almost half of respondents reported experiencing stigma from providers, exacerbating their experience of loss. Positive encounters with care providers were marked by timely, individualized, and compassionate care. Families indicated that improvements in care could be made by providing information and explanations, discharge and follow-up instructions, and through discussions about available supports.

**Conclusions:**

Healthcare professionals can make a positive difference in how loss is experienced and in overall well-being by recognizing the impact of the loss, minimizing uncertainty and isolation, and by thoughtfully working within physical environments often not designed for the experience of loss. Ongoing supports are needed and should be tailored to parents’ changing needs. Prioritizing access to specialized education for professionals providing services and care to this population may help to reduce the stigma experienced by bereaved families.

## Background

Pregnancy and infant loss has a pervasive impact on childbearing families, communities, and health systems. After a loss, families may experience intense grief, sadness, shame, guilt, regret, anger, and stigma, both in the short and long-term [1–12]. They may also experience other life-altering changes, such as changes to relationships, employment, mental health, psychosocial needs in subsequent pregnancies, and personal core and spiritual beliefs [2–4, 10, 13–19]. Many families experience isolation from and changes to their normal support networks, [1, 3, 5, 20–22] often in relation to insensitive comments from others [5, 23, 24]. Families commonly report that their loss is unacknowledged or minimized by their families, social circles, healthcare providers and community [1, 2, 4–7, 17, 19, 25–29]. Prior to this survey, little was known about how families in Ontario accessed and experienced existing perinatal bereavement care and supports, and their thoughts on system gaps and needed improvements.

Ontario is a Canadian province with over 13.5 million residents and approximately 141,000 births annually [30]. Ontario has universal healthcare meaning residents who qualify can access healthcare services in the community and hospital, including primary care, obstetrical, and emergency health services. However, access can be limited by a number of factors including socioeconomic location, gender, immigration status, and geographical location [31]. In Ontario, early pregnancy care (until approximately 12 weeks gestational age) is often provided by a person’s primary care provider (family physician or nurse practitioner), midwives, or in the case of complications, emergency care providers. Family physicians, midwives, nurse practitioners, and obstetricians provide primary maternity care and postpartum follow-up to approximately six weeks post-birth. After this timeframe, health and medical care is often resumed by a person’s primary care provider. Ongoing infant and child healthcare is mainly provided by family physicians, nurse practitioners, and pediatricians.

### Loss statistics in Ontario

Tens of thousands of families in Ontario are impacted by pregnancy and infant loss each year. In Ontario, between April 1, 2016 and March 31, 2017, there were approximately 38,665 recorded visits to emergency departments in Ontario related to pregnancy losses (under 20 weeks gestational age) and their associated complications [32], representing approximately 106 visits to emergency departments every day, or one family every 14 min. Between 2012 and 2016, there were 6904 reported stillbirths [30] and 3283 reported infant deaths in Ontario [33], meaning that one family is impacted by stillbirth every six hours, and one family is impacted by infant death every 13 h in Ontario.

### Bill 141 and PAIL network

In December 2015, Bill 141: The Pregnancy and Infant Loss Awareness, Research, and Care Act was enacted by the Legislative Assembly of the Province of Ontario into law [34]. Bill 141 is the first time any province in Canada has succeeded in passing legislation that mandates research and analysis on pregnancy loss and infant death that assists those who experience such loss and that informs the establishment or expansion of programs related to such loss. Bill 141 was intentionally broad in mandate, focusing program analysis and expansion related to all experiences of pregnancy and infant loss, regardless of gestational age. This groundbreaking piece of legislation has allowed for the issues surrounding pregnancy and infant loss to come to the forefront and begin receiving the attention and government support so desperately needed to evoke change.

As a result of Bill 141, the Pregnancy and Infant Loss (PAIL) Network at Sunnybrook Health Sciences Centre in Toronto, Ontario, Canada, has the funded responsibility, via the Ministry of Health and Long-Term Care, to provide free peer support services to bereaved parents and their families across Ontario, regardless of the nature of their loss, and has as their mandate the improvement and expansion of support services in Ontario. The purpose of this survey was to collect and report aggregate data on people’s experiences of perinatal bereavement supports in Ontario, and to inform the development of a strategic plan by PAIL Network to improve and expand services provided across Ontario, as well as training and support for healthcare and service professionals. The questions that guided our research study were:

“What are the experiences of parents at the time of pregnancy or infant loss with respect to available or needed supports?” and,

“How do families perceive the support provided by healthcare professionals at the time of their loss?”

The information gained from the survey identified practices for supporting people who experience pregnancy and infant loss. PAIL Network is committed to utilizing the voices of bereaved parents in the development of policy and programming, and providing resources and services to people in Ontario.

## Methods

A cross-sectional online survey was conducted in order to gather data about people’s experiences of the loss of a pregnancy or the death of an infant and their thoughts on service and system gaps in Ontario, Canada. Ethics approval was received from the Sunnybrook Health Sciences Centre Research Ethics Board (Approval Number: 443–2016). Contact information for PAIL Network was provided at the start and end of the survey in case participants became distressed in the process of completing the survey.

### Sample

Using purposive sampling, people who lived in Ontario and had a pregnancy loss at any gestation of pregnancy (i.e. the person who carried the pregnancy or their intimate partner(s)), or who had experienced the death of an infant, were eligible to complete the survey about their experiences with the healthcare system and community supports at the time of the loss and during follow up. If participants had experienced more than one pregnancy or infant loss, they were asked to focus on only one loss when completing the survey. Participants were asked about the care and supports they received and to identify gaps or improvements needed. In order to fulfill PAIL Network’s provincial mandate to better plan for the creation of needed supports in Ontario, participants were excluded if they did not live in Ontario at the time of their loss.

Advertisements inviting people to participate were placed on the public Bill 141 Facebook page and PAIL Network website, with a link to the survey, information about the purpose of the study, and contact details for the primary investigator and the chair of the research ethics board for questions related to participation. No identifiable information was collected from respondents. Informed consent was implied through independent completion and submission of the survey. The survey was available for completion online for two months during January and February 2017.

### Survey

The anonymous, web-based survey was designed following a review of the perinatal loss literature and discussed amongst the researchers, which included clinicians and a person with lived experience of pregnancy loss. The draft survey was initially reviewed by a person with expertise in online survey development, and minor revisions were made based on her feedback. The survey was then piloted with a group of women (*n* = 5) with lived experience of loss (miscarriage, stillbirth, neonatal death, infant death) accessed through Sunnybrook Health Sciences Centre. These bereaved parents were asked about their thoughts on the length, content, ease of online completion, and wording of the survey and whether they were able to express what they wanted to express. Minor survey revisions were made based on their feedback. The final survey included 59 closed-ended multiple choice questions (Likert, partial, and unordered) that focused on care and supports discussed or accessed both at and after the time of the loss, as well as current gaps in care and services and the experience of a pregnancy after loss. The format of the final survey included skip-logic such that respondents were directed through different paths based on their responses. In addition, one free-text question was included to allow respondents to express any thoughts or experiences considered important that they were unable to express in the rest of the survey and to further describe their experiences in their own terms. Allowing respondents to describe their experiences in their own terms can illuminate elements of a complex issue and can also function to redress the power imbalance between researchers and participants by giving voice to the respondents’ opinions in a way that is unconstrained by the researchers’ agenda [35, 36].

### Analysis

Descriptive statistics were used to analyze the multiple-choice survey questions. The responses were summarized numerically by Survey Monkey, and then placed in tabular format [37]. For the open-ended question, a qualitative inductive approach using thematic analysis was used as a means of condensing raw textual data into categories and themes to attain a more in-depth understanding of the experiences described within the participant responses [38]. Thematic analysis is seen as a flexible approach for summarizing key features of a large data set and for examining the perspectives of different research participants, highlighting similarities and differences, and generating insights [39]. Thematic analysis was undertaken through a systematic process that included close reading of the raw data, coding, and developing themes related to families’ experience of loss. Data extracts for each theme were considered to determine whether the themes accurately reflected the voices of participants. Peer debriefing and reflexive writing throughout the process were employed to deepen engagement with the data and establish credibility [39].

## Results

In total, six-hundred and seven people responded to the survey between January and February 2017. Respondents were removed from analysis if the loss took place outside Ontario (*n* = 5) or they did not progress past question 3 (*n* = 6), leaving 596 responses for further analysis. Out of the 596 respondents, 79 did not complete the entire survey; 21 responded to less than 25% of the survey questions. If a respondent did not answer a question, they were not counted in the denominator for calculations. For participants who answered the question about relation to the loss or losses (*n* = 515), 501 had experienced the loss themselves, while 14 reported that their partner had the loss or losses. In total, 269 (52%) respondents completed the open-ended question. Within the total responses, 210 made specific reference to their healthcare provider, and 174 of these references highlighted some negative aspect of their care.

### Demographics

The characteristics of survey respondents are summarized in Table 1. The majority of respondents were female, between 30 and 44 years of age, and White although some people identified as Arab, Black, Chinese, Filipino, or Japanese, First Nations or Metis, Latin American, South, Southeast, or West Asian, ‘other’, or Aboriginal or visible minority not included elsewhere (*n* = 73 [13.6%]). Participants mostly spoke English as their primary language, although a small number identified French, Aboriginal language, Chinese (Cantonese), Portuguese, Punjabi, Russian, and Spanish as their primary language (*n* = 13 [2.5%]). For participants who responded to the question asking about their gender (*n* = 515), 13 people identified as male, 495 identified as female, and 7 identified as other. Annual household incomes ranged from less than $5000 to over $150,000, with 45% of respondents having annual household incomes greater than $100,000. Eighty-eight percent of respondents had attended college or university.

**Table 1:** Demographic characteristics of participants. (*N* = 596; some did not respond to all questions, some selected > 1 ethnic groups)

Characteristic		n (%)
Gender		
	Male	13 (2.52%)
	Female	495 (96.12%)
	Other	7 (1.36%)
Age		
	Under 30	60 (11.65%)
	30–39	354 (68.74%)
	40–49	86 (16.70%)
	Over 50	15 (2.91%)
Ethnic group		
	White	452 (87.77%)
Primary language		
	English	501 (97.28%)
Household Income		
	Less than $100,000	220 (42.72%)
	Over $100,000	235 (45.63%)
	Prefer not to answer	60 (11.65%)

### Timing of loss

Six-hundred-and-seven initial respondents reported having experienced a total of 1625 losses, an average of three losses per respondent. Over half of these losses (883) occurred during the first trimester. We asked participants who had experienced more than one loss to respond to the full survey thinking about only one of their losses (Table 2). Following Canadian definitions, timing of loss options to select included:

- Miscarriage (pregnancy loss under 20 weeks gestational age)
- Stillbirth (loss over 20 weeks gestational age)
- Neonatal death (death of baby within 28 days of life)
- Infant death (death of baby after 28 days of life, but within one year)
- Pregnancy termination for medical or other reasons

**Table 2:** Timing of loss (*N* = 596)

		n (%)
Timing of loss		
	Miscarriage or other pregnancy loss under 20 weeks gestational age	330 (55.36%)
	Stillbirth	162 (27.18%)
	Neonatal death	47 (7.89%)
	Infant death	18 (3.02%)
	Pregnancy termination for medical or other reasons	39 (6.55%)
Timing of experience		
	Less than 3 months ago	44 (7.38%)
	3–6 months ago	41 (6.88%)
	6–12 months ago	65 (10.91%)
	1–5 years ago	298 (50.00%)
	5–10 years ago	86 (14.43%)
	More than 10 years ago	62 (10.40%)

Fifty-five percent of respondents thought about a miscarriage (or other pregnancy loss under 20 weeks gestational age) when they completed the survey (*n* = 330). Fifty percent of the losses remembered by the respondents took place one to five years ago. Other responses included in the last three months (7%) and more than ten years ago (10%).

Care providers at the time of the loss are summarized in Table 3. Individuals who were already receiving antenatal care were receiving this care from family physicians, obstetricians or maternal fetal medicine specialists, nurse practitioners, or midwives. Twelve percent of respondents (or their partners) were not yet registered with an obstetrical provider for pregnancy care at the time of their loss.

**Table 3:** Care providers (*N* = 596)

		n (%)
Receiving pregnancy care at the time of loss		
	From a Midwife	121 (20.30%)
	From a Family Physician	214 (35.91%)
	From a Nurse Practitioner	20 (3.36%)
	From an Obstetrician	193 (32.38%)
	From a High Risk Physician (Maternal Fetal Medicine Physician)	79 (13.26%)
	Not receiving pregnancy care at the time	69 (11.58%)
Medical attention for participant or partner at the time of loss because of loss		
	From Primary Care Provider	189 (31.71%)
	From Primary Pregnancy Care Provider	198 (33.22%)
	From Emergency Response	33 (5.54%)
	From Emergency Department	215 (36.07%)
	From a Hospital Labour/Delivery Unit	210 (35.23%)
	From an Early Pregnancy Loss Clinic	27 (4.53%)
	From a Fertility Clinic	31 (5.20%)
	From a Neonatal Intensive Care Unit or Pediatric Unit	0 (0.00%)
	From a Palliative Care Team	0 (0.00%)
	Did not receive medical attention or care at time of loss	30 (5.03%)
	Other	42 (7.05%)

At the time of their loss, participants received care from their primary care provider, Emergency Medical Services (EMS), or in a birthing unit or the emergency department, as well as walk-in clinics, early pregnancy loss clinics, operating rooms, perinatal hospice programs, and fertility clinics. For this question, respondents were able to select all applicable care provider options. Totals indicated that many people accessed care at multiple points and from multiple professionals during their loss. Five percent indicated that they did not seek any medical attention at the time of their loss.

### Time of loss: care received from healthcare professionals

Evaluation of the care received at the time of the loss is included in Table 4. Of the participants who stated that they received care from a healthcare professional at the time of their loss, 47% of participants responded ‘yes, definitely’ when asked if they were treated with kindness and respect.

**Table 4:** Evaluation & experiences of care

	Question, phrase, or option	n % - options
At the time of loss		
	Treated with kindness & respect	266 (47%) - Yes, definitely
	Given information	157 (28%) - Yes, definitely
	Felt supported	195 (35%) - Yes, definitely
	HCP had skills needed	175 (31%) - Yes, definitely
	Not given any discharge or follow up instructions	129 (23%) – selected this option
	Experienced stigma	136 (24%) & 139 (25%) - Yes, definitely & Yes, somewhat
Follow-up care		
	Offered appointment (regardless of whether attended)	320 (55%) – selected this option
	Treated with kindness and respect	261 (69%) - Yes, definitely
	Felt supported & cared for	230 (60%) - Yes, definitely
	HCP had skills needed	194 (51%) - Yes, definitely
	Experienced stigma	31 (8%) & 71 (19%) - Yes, definitely & Yes, somewhat

When asked if they were given the information and explanations they needed from healthcare providers, 28% responded ‘yes, definitely’. When asked about feeling supported and cared for by healthcare providers, 35% of participants responded ‘yes, definitely’ and 31% of the same participants responded ‘yes, definitely’ when asked if the healthcare providers had the skills needed to respond to their needs. When asked about being given discharge or follow-up instructions from healthcare providers, 23% indicated they were given no instruction, while 8% indicated they did not remember if they were or not. When people received discharge or follow-up instructions, verbal (55%) and written instructions (29%) were most common.

When asked if they experienced stigma from healthcare professionals, defined in our study as ‘insensitive comments about how you should feel, grieve, or experience the loss that you did not agree with, or care that did not recognize the significance of your loss or your need for support’, [40, 41] 49% of participants responded ‘yes’.

### Follow-up appointment

Evaluation of the care received at the time of the loss is included in Table 4. The majority of participants (67%) had a follow-up appointment with a healthcare professional after their loss (they either were offered one or made one themselves). Fifty-one percent of those who attended a follow-up appointment were offered an appointment at or around the time of their loss, while the other 16% made the follow-up appointment themselves. At the follow-up appointment, 69% responded ‘yes, definitely’ when asked if they were treated with kindness and respect, and 60% responded ‘yes, definitely’ when asked if they felt supported and cared for by the healthcare provider. Slightly more than half (51%) of all participants who had a follow-up appointment responded ‘yes, definitely’ when asked if the healthcare provider had the skills needed to respond to their needs. At the follow-up appointment, 27% of the total participants reported experiencing stigma about their loss from the healthcare provider.

### Sources of support

The most common sources of support at the time of the loss included partners (82%), friends (69%), parents (63%), and other family members (44%). Employers (35%) and co-workers (28%), as well as online support (26%) and other bereaved parents (25%), were also listed as sources of support. When asked to choose their most important source of support, 43% of participants selected their partner. Healthcare professionals were reported as sources of support to families as well. These included family physicians (40%), hospital nurses (33%), obstetricians (33%), and midwives (16%). When asked about the type of support provided by a healthcare professional, the most common responses were medical or physical support (64%), emotional support (55%), and informational support (49%).

Participants were asked about the types of formal and informal sources of support that they were told about around the time of their loss. Almost half of all respondents (45%) were not told of any sources of support. The most common sources of support that participants were told about were written supports (24%), PAIL Network support groups or local community peer support groups (21% respectively), and professional counseling (20%).

Sources and obstacles of support are presented in Table 5. Participants were asked if they accessed formal or organized supports around the time of their loss, and 58% reported they had done so. The most common ways to access support included ‘in person (64%), ‘Facebook’ (45%), ‘internet or other loss support websites’ (45%), ‘books or written resources’ (45%), ‘phone’ (26%), or ‘email’ (18%). In person support, books and written resources, and online support were most rated as ‘very helpful’ (45, 30, and 29% respectively). Facebook, Twitter, other social media, and the internet, when combined, were the most helpful sources of support across all groups.

**Table 5:** Support

	Question, phrase, or option	% - options
Support Accessed		
	Easy to access support	22% - Very easy & Somewhat easy
	Accessed support	58% - selected this option
	Accessed peer support	78% - selected this option
	Able to get needed support	23% - Yes, definitely
	Need for support changed over time	76% - selected this option
	How much support is in your community?	15.5% - More than enough & Enough
Obstacles faced		
	I didn’t know what supports were available	65% - selected this option
	No local supports available in my community	33% - selected this option
	Stigma or silence about pregnancy and infant loss	23% - selected this option
	Available supports did not meet my personal emotional needs	20% - selected this option
	I was feeling unwell and unable to access supports at the time	18% - selected this option
Efficacy of peer support (Top 6 reasons)		
	Knowing that I was not the only person to have experienced pregnancy or infant loss	89% - selected this option
	Being able to hear about other people’s experiences with pregnancy or infant loss	83% - selected this option
	Being able to talk openly about my loss experience	78% - selected this option
	Being able to share my story with others who have experienced pregnancy or infant loss	74% - selected this option
	I felt less alone or isolated	71% - selected this option
	Being able to talk openly about my baby	70% - selected this option

When asked about how easy it was to access support around the time of their loss, 6% of total respondents selected ‘very easy’ and 18% selected ‘very difficult’. The survey also asked participants about the obstacles they faced when trying to access formal or organized supports (Table 5). The most common obstacles included not being aware of resources or that there were no resources available.

### Support – changing needs over time

Seventy-eight percent of participants indicated that over time, the type of support they needed changed. Reasons for this changing need, selected from an a priori list with an additional option for open text (‘other’ option), included relying more on themselves (36%), needing more formal professional one-on-one support (33%), and still needing support, but not as often as before (32%). Thirty-one percent indicated that they relied more on family and friends for support over time. When asked about whether or not they were able to get the support they needed over time, 23% of total participants responded ‘yes, definitely’.

### Peer support

Participants who indicated that they accessed support around the time of their loss (58% of total respondents) were then asked specifically if they accessed peer support. Peer support, defined as ‘support provided by other bereaved parents’, was accessed by 78% of these respondents (Table 5). Participants who responded ‘yes’ to accessing peer support were asked about what was useful or helpful about this type of support (Table 5). One percent indicated that peer support was not useful or helpful. When asked if there was anything that they did not like or found challenging about the peer support they accessed, the most common responses, selected from an a priori list with an additional option for open text (‘other’ option), were ‘no’ (35%), ‘location of group was not convenient’ (18%), ‘not enough support for men’ (16%), ‘other’ (15%), and ‘timing of group was not convenient’ (13%).

### Community supports

When asked about how much support was available for families experiencing pregnancy or infant loss in their local community, 84% of total respondents selected either ‘not enough’ or ‘none’. Key areas of concern for participants for families experiencing loss in their communities, selected from an a priori list with an additional option for open text (‘other’ option), are listed in Table 6, and included reducing stigma and silence about perinatal loss, automatic referrals to bereavement or counseling supports, more public awareness about the frequency and impact of pregnancy and infant loss, and better follow-up by healthcare professionals.

**Table 6:** Key areas of need or concern

	Question, phrase, or option	% - options
Top 10 areas		
	Reducing stigma or silence about pregnancy and infant loss	69% - selected this option
	Automatic referral to bereavement or counseling supports for families after loss	66% - selected this option
	More public awareness or education about the frequency and impact of pregnancy and infant loss	62% - selected this option
	More public awareness or education about existing supports	59% - selected this option
	Better follow-up by healthcare providers for families after loss	56% - selected this option
	Better training for healthcare providers who care for families experiencing pregnancy and infant loss	55% - selected this option
	Early pregnancy loss clinic	55% - selected this option
	Availability of skilled and compassionate healthcare providers	53% - selected this option
	Better mental health supports	51% - selected this option
	More information in general about pregnancy and infant loss	51% - selected this option

### Thematic analysis

Four themes were identified from qualitative analysis of the open-ended question: a) isolation and exclusion, b) recognizing and responding to loss, c) accessing information and supports, and d) making a difference.

### Isolation and exclusion

Many respondents described isolation and exclusion as significant factors influencing their experience of loss, with one respondent explaining, “It just felt like once I wasn’t pregnant anymore, I didn’t matter to anyone.” *(Miscarriage/early loss)* Stigma led to further isolation. One person reported that they “faced stigma and felt embarrassed like I should hide my losses and I was responsible.” *(Miscarriage/early loss, stillbirth)* Another wrote that having waited to disclose the pregnancy only increased their sense of isolation and the stigma surrounding loss, noting, “We hadn’t told anyone that we were expecting so suffered alone and in silence.” *(Miscarriage/early loss).*

The uniqueness of the loss for each person meant that many felt unwelcome within some groups or that they did not belong, leading to feelings of exclusion and inability to access desired supports. As one person reported, “I made the mistake of attending an open loss group and was told by another group member that my loss wasn’t the same as theirs because I could have another baby but she couldn’t.” *(Miscarriage/early loss, stillbirth)* Exclusion was also experienced by an adoptive mother, who explained, “I did not physically birth him. But he was mine. I felt like I couldn’t go to any support groups. Like I wouldn’t fit in.” *(Infant death)* There was also a perceived gap in supports available for people who undergo fertility treatments after a loss. As one respondent explained, “a lot of fertility patients have suffered multiple miscarriages and receive fertility support but never deal with the actual emotions and trauma of the miscarriages.” *(Miscarriage/early loss, stillbirth).*

Fathers and same gender families also experienced feelings of exclusion. The impact of the lack of support on relationships was evident in another person’s comment that they were watching their husband “fade away” as he felt he had nobody to talk to. *(Neonatal death)* Heteronormativity exacerbated feelings of isolation for LGBTQ2S (lesbian, gay, bisexual, transgendered, queer, two-spirited) families, as noted by one respondent who said, “I often felt isolated because of how gendered the support groups are. There are many queer families suffering from pregnancy and infant loss and it seems there isn’t a place for the partner”. *(Miscarriage/early loss, stillbirth)* Partners also wished for a more inclusive approach to care. One father explained that,

“As a male, I understand that priority in pregnancy and baby losses is given to mom. However, I really hope that compassionate and integral family care can be offered to all family members. There is a lot of stress for fathers too (or same gender partners), who on top of having their own grief are expected to ‘stay strong’”. *(Miscarriage/early loss, stillbirth).*

### Recognition and response to loss

The difference in the meaning of the loss between families and healthcare professionals influenced recognition and response. This was evident in the terminology used and attempts to reframe the loss in technical terms. One person explained, “I was telling them about my baby, which I delivered at home, and was calcified, they interrupted me and called it a fetus.” *(Miscarriage/early loss).* Another person reported that, “after telling me I was having a miscarriage my GP asking why I was crying…I said I wanted that baby…and then he said it was not a baby it was a fetus.” *(Miscarriage/early loss).*

Comments from healthcare professionals such as, “many women go through this”, “you can try again”, and “it wasn’t meant to be” *(all miscarriage/early loss)* had an impact on the respondents, reflecting a lack of compassion and recognition of the impact of the loss. Many respondents perceived that their devastating loss was treated like a routine event by healthcare professionals, with one respondent noting, “Doctors who see this tragedy every day, need to be reminded that this is a first for us.” *(Neonatal death)*. As another person explained, “The doctor who performed my D&C was very insensitive, treating my miscarriage as a normal, everyday event. It was the most tragic thing that ever happened to me”. *(Miscarriage/early loss).*

The need for healthcare providers to attend to the power of language used at the time of loss was evident, as one respondent noted,

“During my second loss, I was under the care of a very insensitive [healthcare professional]. After that happened, I left her care because I couldn’t see going back to her after that. She kept referring to my loss as a ‘spontaneous abortion’ which I’m sure is clinically correct, but even after I asked her to stop using the term she continued to do so. It made my appointments very painful mentally and I really dreaded them. Health care practitioners need education on how to speak to people who have been through loss with sensitivity.” *(Miscarriage/early loss).*

The uncertainty of ‘knowing’ something was wrong, not being believed, and waiting for confirmation of the loss, along with lack of information, emotional support, and anticipatory guidance added to the distress experienced by the respondents. One person who had a termination at 23 weeks explained, “I wasn’t prepared for a full blown labour …we didn’t know we’d have to arrange a funeral or what our baby would look like or what decisions we’d have to make…”*.* Another noted, “We were told to anticipate “a heavy period”, when in reality we were experiencing the labour and delivery of a small, dead, baby.” *(Miscarriage/early loss).*

The environment of care shaped recognition and response to perinatal loss. Many people went to emergency rooms where they were not seen as urgent or important and where they perceived that healthcare professionals lacked the training, knowledge, and sensitivity to respond appropriately to their loss. As one person reported,

“The ER nurse told me ‘Get control of yourself or I won’t talk to you… If you want to see someone you’ll have to wait.’ I went into the ER bathroom and ended up miscarrying there. It was unbelievably awful…I felt judged, ignored, and discarded.” *(Miscarriage/early loss, medical termination of pregnancy).*

Another person described her experience of miscarrying in the hallway of a hospital in a bedpan, explaining, “they left it on the floor and other nurses were saying ‘ewww what’s that’...then I heard them say ‘throw it away’... my dearly loved child ... I cry as I think about it”*.*

One person cited the importance of healthcare professionals' knowledge and awareness in ensuring recognition of loss and access to supports. When making the difficult decision to end a non-viable pregnancy early, one mother was told by a social worker that she was “not eligible” for the late loss group, only to find out later – after emailing the group facilitator – that they were welcome. Another person who experienced a loss at 14 weeks was advised that “statistically” this was not considered to be an infant loss. A respondent who experienced what was described as ‘a chemical pregnancy’ explained that these were not perceived as legitimate pregnancies and babies, noting, “There needs to be a certain level of compassion, understanding, and empathy when discussing these types of losses, particularly for those women who disclose the importance or sadness associated with the loss”*.*

Respondents also described a lack of recognition in the workplace of the impact of perinatal loss. This was evident in the lack of policies to support work absence as well as insensitivity encountered from managers, colleagues, human resources, and insurance companies. They described being “harassed” for taking sick days *(Miscarriage/early loss)* and being told by their employers that they had been on leave “long enough.” *(Stillbirth)* Many described highly unsupportive and uncompassionate workplaces and struggled to explain the significance of their loss in order to take time off. The financial consequences of stigma related to perinatal loss in the workplace were real. One person explained that they almost lost their home due to the severe depression that occurred after their bereavement leave. Another who applied for short term disability believed that this impacted future promotions while one person who was a contract worker was laid off upon their return and they were unable to pursue legal challenges due to their contract status.

### Accessing information and supports

Responses indicated high variance in the services available, including knowledgeable caregivers and written material, further compounding the loss experience. A frequently cited concern of respondents was wishing they had had more support at the time of their loss, and guidance about what to do. As one person explained,

“We were so shocked we needed guidance on next steps and our options and we weren’t thinking straight. I wish I was told I could take pictures of him, that I could bathe him, that I could have more time with him…now I am filled with regret that I didn’t spend more time with him and take more pictures of him. I wish a doctor/nurse/social worker told me I could do this as I know I would have said yes if given the option”. *(Stillbirth).*

The lasting impact of lack of access to this kind of support was also captured by one person who stated,

“I’m crying as I write this, because so many of these things my husband and I would have loved to have done and had the memories doing them. It was too hard at the time for us to think straight.” *(Stillbirth).*

Many respondents living in rural areas noted the lack of availability of resources and knowledgeable care givers in their communities. In these cases, transportation and the need for child care prevented families from accessing much needed supports. In many locations, even when resources were available, families encountered barriers when trying to access these resources. Some reported that they were given outdated pamphlets and phone numbers while others were never offered information or support or counseling options. Follow-up by healthcare professionals was also cited as a concern, with one parent noting that, “I had called the social worker and left a message but nothing was returned. I felt as though I was left out in the dust.” *(Stillbirth)* The availability of spiritual care and legal advice was also noted as an important element of support that was often unavailable. One person who was unable to access spiritual care reported that nothing was offered, stating, “I needed that support, but it wasn’t offered in any booklet, or pamphlet ... the chaplain wasn’t asked to come, and there was no pastoral care information at all.” *(Miscarriage/early loss).*

### Making a difference

Respondents indicated that the actions of healthcare professionals could make a positive and lasting difference in the experience of loss. These actions consisted mostly of small gestures from providers in a range of disciplines that were integrated into care. One parent noted,

“The care I received in hospital was amazing. The nurses were there to talk, help us after he was born and the following day with pictures and allowing us to spend more time with our son before I had to leave.” *(Stillbirth).*

It was often the little things that made a difference, as in the case of one person who explained that,

“I was thankful for my nurse who came in after to do bloodwork because she took two minutes to give me a hug, a cloth to clean my face, some clean pads and said ‘I’m so sorry for your loss’. Then she did what she needed to do. A little compassion goes a long way.” *(Miscarriage/early loss).*

Another noted,

“Some of the nurses for the quick minute I got to speak to them shared their experiences and it happens so often and yet no one talks about it. It was nice for them to share and it made me feel better.” *(Miscarriage/early loss).*

Respondents appreciated any contact from care providers that let them know they had some understanding of what they were experiencing. For example, one person said,

“I was so impressed that my family doctor called me directly over the phone to see how I was doing… (She) had never called me before but she clearly understood that this situation was different and that I would need someone to talk to and benefit from hearing from her directly so that I could ask questions.” *(Miscarriage/early loss).*

Another reported that, “My midwife...made sure that I understood the process, she made sure I kept my baby overnight and then she followed up with me weekly for 6 weeks afterward.” *(Stillbirth).*

The power to make a positive difference in the experience of loss was evident in one person’s story who had had a Caesarean section and was encouraged to be involved in planning her baby’s funeral. “It was therapeutic…my husband and I both got to see our child leave the morgue. I always have appreciated that our wish to do this was taken seriously. For that I am forever grateful.” *(Neonatal death).*

## Discussion

This study reports on families’ experiences of all types of pregnancy and infant loss. Specific areas of focus for this discussion include stigma and the meaning of loss, the context of care, and supports.

### Stigma and the meaning of loss

Consistent with other studies [19, 40, 42–45], respondents described experiences of disenfranchised grief, whereby losses are not legitimized by health and service professionals, families, and communities, as a source of distress. The insensitive language of loss, for example, the use of ‘fetus’ or ‘abortion’ by healthcare professionals, contributed to a sense of isolation. Our findings continue to shed light on the difficulties many families faced after pregnancy or infant loss, and how families live in the world after this experience – as ‘othered’. This study highlights the commonality of the experience of isolation, regardless of the time and type of loss, and the desire to have the meaning of loss recognized by healthcare professionals. If healthcare professionals minimize or misunderstand the impact of loss, even unintentionally, they may be less likely to recognize families’ needs for anticipatory guidance and referrals to available resources and supports.

Our findings surrounding the frequent impact of stigma and silence surrounding pregnancy and infant loss has also been described in the literature [5, 12, 14, 22, 24, 27, 41, 45–50]. As stigmatization can be enacted by thoughtlessness or insensitivity, paying attention to the words used and personal biases around loss, which have the potential to either intensify suffering or affirm the meaning of the loss for families, may be a means by which professionals can avoid perpetuating feelings of stigma and isolation [40, 51]. Understanding the extent to which stigmatization impacts grief and healing should be a priority in care provision and future research.

#### Healthcare professionals and the context of care

Our finding that families did not always receive the quality of care that they desired has been reported elsewhere [2, 4, 7, 8, 17, 20, 25, 26, 28, 29, 52–56]. Bereavement care is challenging and difficult work for care providers, who are often managing competing demands and resource limitations [5, 57]. A lack of clear clinical guidelines may complicate the provision of skilled and compassionate care, as may a lack of formal education for most professionals [15, 20, 28, 46, 54, 58–61]. Individual risk factors such as lack of experience, personal feelings of inadequacy, and frequent exposure to loss may cause some professionals to emotionally disengage and distance themselves from care. There is a need to build supportive and responsive communities of practice for professionals who witness the suffering of families in their everyday practice. As identified elsewhere [15, 21], our findings support the need to develop bereavement practice guidelines or care pathways that may help to minimize the wide variation in care that families receive from the healthcare system and healthcare professionals, both at the time of and after the loss. At the same time, families also value personalized supports.

While the environment of care influenced the experience of loss, some families described professionals who were able to overcome inhospitable contexts and have a positive impact on the loss experience, regardless of their workplace setting. While the lack of resources and time may be constraining factors, the findings in our study, supported elsewhere, suggest that families placed importance on a provider’s caring manner and a willingness to communicate [21, 56, 62]. Despite ongoing challenges, professionals can make a positive impact with the care they provide.

Consistent with previous studies [10, 14, 15, 20, 21, 28, 56, 63], communication failures created barriers to acceptable care for families. Families in our survey indicated that improvements in care could be made by providing information and explanations, discharge and follow-up instructions, and through discussions about available supports. Information and supports should be culturally sensitive and available in multiple languages. Opportunities exist to better develop the communication skills of healthcare professionals. Knowledge translation strategies for moving feedback about quality care into practice is an area for future research.

### Support

Although families accessed different types of care at multiple points during their loss, almost half were never told about available supports. Despite evidence that peer support is valued by families, [1, 20, 21, 26, 44, 64] we found that many families were still not told about or able to access peer support services. Echoing other literature, [65–68] families indicated peer support was helpful because it reduced isolation, allowed people to share about their babies and experiences, and provided opportunities to listen to others talk about their babies. As discussed elsewhere [56], partners, family members, and healthcare professionals were identified as sources of support. When interacting with families, we encourage professionals to assume that a discussion about available resources and supports has not already happened, and to initiate this conversation.

Families in our survey identified support needs that continued long past the loss event. An unanticipated finding was how often families reported that their needs for support changed over time, indicating a need for diverse types of support to meet the ongoing needs of families. This has implications for the anticipatory guidance provided by healthcare professionals and for program planning to ensure community resources reflect changing needs.

Our findings align with the work of others that have highlighted the need to invest in better supports for families, as well as the need to clearly identify supports to families [14, 19, 22, 26, 45, 53, 55, 69]. We are concerned about the long-term implications when the need for support is not recognized, families are not told about supports, and access to supports is limited. Attempts to better quantify and inform health policy about the tangible and intangible costs of perinatal loss, such as impact on the health of the partner, financial stress, and work-related lost productivity, have been undertaken by several researchers [5, 6, 13, 70–74]. More research into the long-term consequences of not getting support following loss for the individual, their partner, and the community is needed. The development and provision of family-identified supports should be a continued focus of government, health systems, and communities.

### Strengths and limitations

This study collected information via an internet survey, which had the potential limitations of self-selection bias, sampling bias, and coverage bias [75]. Most respondents to our survey would have seen the invitation to participate in two main online locations where they were already actively involved, and thus already connected to the perinatal bereavement community. People without online access were excluded from this study, as were those who could not complete a survey in English. Limitations of this study also included a lack of diversity in race, gender, and economic backgrounds of the individuals who participated in the survey, which limits the generalizability of our findings. While thematic analysis of the open-ended question facilitated understanding of the experiences of families in their own words, having only one open-ended question also limited the extent to which we were able to more deeply understand, qualitatively, the lived experience of perinatal loss [35].

In our analysis, we focused on reporting responses that clearly indicated that good, quality, and respectful care was received, but acknowledge that this expectation and interpretation for acceptable provision of healthcare may be debated. For example, when asked if at the time of their loss they felt treated with kindness and respect by the healthcare provider(s), we highlighted when respondents selected ‘yes – definitely’, and did not highlight responses of ‘yes – somewhat’ or ‘no’, as we perceived both responses to indicate a gap or need for improvement. As reported elsewhere [19, 21, 53, 56], several respondents indicated that during their loss, kind, skilled, and respectful care was inconsistent, sometimes depending on the professional or environment of care. We acknowledge that fixed survey questions result in a loss of ability to capture subtleties and to address variations in care that exist in the ‘real world’.

Strengths of this study were the large number of participants for qualitative data collection and that this was the first study of its kind surveying a large population of families experiencing all types of pregnancy and infant loss to be conducted in Canada. The use of qualitative and quantitative data was a strength that contributes to our understanding of the needs of this population.

## Conclusion

This study has the potential to raise awareness and provide strategies to improve care for families experiencing the loss of a pregnancy or the loss of an infant. As one participant expressed, “Our experiences do not disappear or reset with a new health concern or pregnancy. Loss parents need to be treated with empathy and compassion always so they can trust their healthcare professionals the next time they are patients”.

Quality in healthcare is shaped by the experiences and engagement of patients, families, caregivers, and professionals. Families’ responses indicated a clear need of and directions to improve care for those experiencing the loss of a pregnancy or the death of an infant in Ontario. Healthcare professionals can make a positive difference in how loss is experienced by recognizing the impact of the loss, providing compassionate care through the use of sensitive language and physical supports, minimizing uncertainty and isolation, and by sensitively working within physical environments often not designed for experiences of loss. Access to specialized education should be prioritized for professionals providing services and care to this population.

## Abbreviations

D&C: Dilation and curettage
EMS: Emergency Medical Services
ER: Emergency Room
GP: General practitioner
HCP: Healthcare providers
LGBTQ2S: Lesbian, gay, bisexual, transgender, queer, 2-Spirit
N: Population size
n: Sample size
PAIL Network: Pregnancy and Infant Loss Network
